# Supplementary material for: A Case-Based Active Learning Session for Medical Genetics Resources
Source: MedEdPORTAL. 2021 Apr 1;17:11135. doi: 10.15766/mep_2374-8265.11135 (PMC8015619; doi:10.15766/mep_2374-8265.11135)
Supplement: Supplementary file 1 — Syllabus Introduction.docxStudent Preclass Hands-on Exercise.docxSession Timetable.docxDidactic In-class Discussion.docxStudents In-class Activity.docxFaculty Preclass Hands-on Exercise.docxFaculty Guide In-class Activity.docxPostsession Survey.docx [file mep_2374-8265.11135-s001.zip › A. Syllabus Introduction.docx]

**Evidence-based and Lifelong Learning in Human and Medical Genetics**

**Introduction**

Rapidly expanding knowledge of human genetics and genomics is significantly increasing the importance of genetic diagnosis in today’s medical practice. Differential diagnosis includes interpretation of sequence variants; patient management may be informed by the interpretation of genetic testing results; and, communicating test results frequently includes both patients and their family members. As part of lifelong, self-directed learning, the ability to identify reliable and current information on genetic conditions will be an essential skill for future clinicians to be able to manage patients with genetic conditions.

The goal of this case-based active learning session is to introduce students to credible, specialized medical genetics resources and to provide them with the knowledge and skills to search for and identify relevant information, in order to support development of the lifelong learning skills that are required for managing patients with genetic conditions.

**Clinical questions suggested to be asked for a patient with a possible genetic condition**

(adopted from Diehl et al. Genet Med. 2015. 17:158-63)

Note: Each question is linked to the tags listed in “Recommended Resources: Medical Genetics Resources”

1. What is the differential diagnosis for these signs and symptoms? (Tag: *Differential diagnosis*)
2. What genes are possibly associated with these features? (Tag: *Genotype-phenotype relationships*)
   - Be aware of “**locus heterogeneity**”. Very similar (or overlapping) pathological conditions could be caused by a mutation in different genes.
3. What allelic variation exists for the gene(s) associated with the patient’s condition? (Tag: *Genotype-phenotype relationships*)
   - Be aware of “**allelic heterogeneity**”. Disease-causing mutations occur at different locations within a single gene (e.g. different amino acid positions, promoter sequence, intron sequence).
   - Depending on the location of the mutation within the gene, severity of the disease can differ, which is termed “**phenotypic heterogeneity**”.
4. What is the optimal test to confirm the diagnosis? (Tag: *Genetic testing*)
5. Are any management guidelines available for this condition, such as consensus guidelines published by professional medical societies? (Tag: *Patient management*)
6. Are there any drugs/exposures to avoid? – need to be aware of genetic polymorphisms that affect drug metabolisms such as CYP450 polymorphisms. (Tag: *Pharmacogenetics*)
7. Where can I find a support group for the patient? (Tag: *Patient advocacy and support*)
8. Are the patient’s relatives at risk? Who should be screened to identify “at risk” individuals in the family? (Tag: *Family counseling*)
9. Are any clinical trials for this condition available? (Tag: *Clinical trials*)

**Pre-Class Assignments**

1. Complete ‘Pre-Class Assignment – OMIM tutorial for single gene disorders’ by submitting answers via the Genetics course website. These are not multiple-choice questions and you will get half credit of the session by completing all five questions of ‘Pre-Class Assignment – OMIM tutorial’. Another half of the credit is based on attending the in-class session. Together, you will get full credit.
2. Review “Recommended Resources” listed below. At the beginning of the in-class session we will briefly review these web resources.

**Recommended Resources: Medical Genetics Resources**

- OMIM (Online Mendelian Inheritance in Man) <https://www.omim.org/>
- *Differential diagnosis, Genotype-phenotype relationships*

This site is a good launchpad for collecting background information on genetic conditions. Hyperlinks to other useful resources are provided on the webpage of each genetic condition under External Links (e.g. GeneReviews, MedlinePlus Genetics, ClinicalTrials.gov). Comprehensive description of clinical presentation (clinical synopsis) and in-depth research on known molecular causes of genetic conditions are presented. With regard to chromosomal disorders, some are listed (e.g. down syndrome), some are not (e.g. Klinefelter syndrome). For chromosomal disorders, it is easier to use MedlinePlus Genetics or MedGen to obtain background information (listed below).

- GeneReviews <https://www.ncbi.nlm.nih.gov/books/NBK1116/>
- *Differential diagnosis, Genotype-phenotype relationships, Genetic testing, Patient management*, *Family counseling*

This site presents comprehensive clinical reviews for genetic conditions of single-gene disorders and syndromes caused by chromosomal micro-deletion/duplication. Very useful for obtaining background as well as foreground information on clinical diagnosis, management, and genetic testing.

- MedlinePlus Genetics <https://medlineplus.gov/genetics/>
- *Genotype-phenotype relationship, Patient advocacy and support, Family counseling*

This is another good launchpad site. MedlinePlus Genetics provides information in plain English (in lay terms) on a large number of genetic conditions (single gene disorders, chromosomal disorders), genes, and genetic terminologies. Information is comprehensive and hyperlinks for other useful sites (e.g. patient support groups) are also provided. A good information source for counseling patients and patient education.

- Medical Genetics Summaries <https://www.ncbi.nlm.nih.gov/books/NBK61999/>
- *Pharmacogenetics*

Important pharmacogenetics variants for variable drug response are summarized. Listing is limited, but essential pharmacogenetics information is found here. Also, ‘Clinical Pharmacogenetics Implementation Consortium (CPIC)” <https://cpicpgx.org/> provides consensus guidelines for pharmacogenetic testing. The most comprehensive pharmacogenetics/genomics information website is <https://www.pharmgkb.org/>.

- Genetic and Rare Diseases Information Center (GARD) <https://rarediseases.info.nih.gov/>
- *Genotype-phenotype relationship, Patient advocacy and support, Family counseling*

GARD provides the public with access to current, reliable, and easy-to-understand information about rare or genetic diseases in English or Spanish, delivered in lay terms. This is an excellent resource to find support for patients and their families.

- ClinicalTrials.gov <https://clinicaltrials.gov/>
- *Clinical trials*

This is a database of privately and publicly funded clinical studies conducted around the world. The website lists completed, on-going, and recruiting clinical trials.

- MedGen <https://www.ncbi.nlm.nih.gov/medgen/>
- *Differential diagnosis, Genotype-phenotype relationships, Genetic testing*

A good launchpad site. OMIM, GeneReviews, Genetic Testing Registry and other clinical genetics sites are also cross-referenced on this website.

- ClinVar https://www.ncbi.nlm.nih.gov/clinvar/
- *Genotype-phenotype relationships, Genetic testing*

This website provides a public archive of reported human sequence variants and their phenotypes. Clinical interpretations of reported sequence variants with supporting evidence (i.e., benign, pathogenic, sequence of uncertain significance) can be found here.

Other useful websites:

- PubMed – biomedical literature <https://pubmed.ncbi.nlm.nih.gov/>
- UpToDate – clinical information <https://www.uptodate.com/>
