# Supplementary material for: A Case-Based Active Learning Session for Medical Genetics Resources
Source: MedEdPORTAL. 2021 Apr 1;17:11135. doi: 10.15766/mep_2374-8265.11135 (PMC8015619; doi:10.15766/mep_2374-8265.11135)
Supplement: Supplementary file 1 — Syllabus Introduction.docxStudent Preclass Hands-on Exercise.docxSession Timetable.docxDidactic In-class Discussion.docxStudents In-class Activity.docxFaculty Preclass Hands-on Exercise.docxFaculty Guide In-class Activity.docxPostsession Survey.docx [file mep_2374-8265.11135-s001.zip › B. Student Preclass Hands-on Exercise.docx]

**Pre-class Assignment – OMIM Tutorial for Single Gene Disorders**

**Case:**

A 25-year-old male patient presents with blurred vision in clinic. On exam, the physician finds that the patient has a lens dislocation in his eye (ectopia lentis). Additional physical exam findings include: very long fingers (arachnodactyly), unusually long arms, and a slight scoliosis. The patient’s overall stature is tall and slender. His face is relatively long and narrow, without other remarkable features. His family history includes a mother who died from a thoracic aortic aneurysm rupture in her 50’s and also had problems in the bones and joints. The physician suspects that the patient may have a genetic condition and would like to investigate if there is a genetic basis for the patient’s condition.

Using OMIM, we will work on the following questions step-by-step.

1. What is the differential diagnosis for these findings?
2. What other symptoms/findings might this patient exhibit?
3. What is the mode of inheritance of each genetic condition?
4. Where are those genes located?

- **Pre-class Hands-on Exercise**

Go to the OMIM homepage at <https://www.omim.org/>

a. Basics of OMIM search:

1. For basic searches, you can enter the terms (e.g. clinical features or phenotypes) in plain language in the search box and then click ‘Search’.
2. When your search returns a large number of results, there are a few ways to refine your search. For example, when the term ‘loose joints’ is used for your search, to refine the search add ‘+’(plus) in front of each word: +loose +joints. Alternatively, you can search for a phrase by adding quotation marks: “loose joints”.
3. Your search retrieves both gene and phenotype as results. They are differentiated by a symbol next to each search result entry: * indicates a gene entry and # indicates a phenotypic entry. A gene entry provides descriptions of a gene and a phenotypic entry provides descriptions of a genetic condition/disease that is found associated with the term you used for the search.

b. The case:

Based on the patient interview and physical exam, what were significant findings? Summarize these findings in medical terms if you can and use them to search OMIM. Use “quotes” around phrases. If you want the term or phrase absolutely contained as part of a search query, use + in front of the search term or phrase.

To begin, try the following search in OMIM. In the basic search box, enter the following search string, and then hit return:

"ectopia lentis" arachnodactyly scoliosis “thoracic aortic aneurysm”

You will probably find that this search strategy retrieves over 1000 results. To narrow the search, add a + symbol in front of each search term or phrase and hit return. Did the number of returned results become more manageable? Do you remember what # or * in front of the ID number indicates?

Look for the first gene entry listed in the search results and open that link in new tab.

How many different phenotypic entries are associated with this gene? (refer to Gene-Phenotype Relationships table on the webpage) The term ***Phenotypic heterogeneity*** is used when mutations occurring at different locations within the same gene result in different phenotypes (different enough to be given different disease names).

Scroll down to TEXT, read ‘Description’ and ‘Cloning and Expression’ sections. Does the function and expression of the protein encoded by this gene provide an explanation for the phenotype of this patient, i.e. the reason why he has clinical manifestations in multiple organs?

Go back to the search result page. On the upper right corner, find “Clinical Synopsis” and click/open this link in new tab. Check the little box in front of each genetic syndrome and click “Compare Selected”.

Now you have a table of the genetic syndromes and their clinical presentations that are candidates for our patient. Let’s answer the questions below.

**Q1. List the names of genetic syndromes obtained in this search.**

**Q2. What additional phenotypic data from the patient interview and physical exam can you use to narrow down possible diagnosis for this patient?**

**Q3. Using the additional phenotype(s) above, which are the most likely candidate diagnoses?**

**Q4. List the genes associated with these candidate diseases for our patient.** (Hint: they are listed in “Molecular Basis” at the bottom of the clinical synopsis table)

The term ***Locus heterogeneity*** is used when similar or overlapping pathological conditions are caused by mutations in different genes at different chromosomal loci.

At this point, you have decided to refer your patient to a genetic counselor. The counselor met with your patient and recommended ordering genetic testing for the patient. The testing result came back as follows. **FBN1: c.6920G>C (p.Cys2307Ser).** Let’s think about what this test result means.

Refer to the bottom row of the clinical synopsis table listing “Molecular Basis”. Here you see the name of the gene associated with each genetic syndrome. Click on the hyperlink for the gene name indicated in our patient’s genetic testing. Now you see representative pathogenic mutant alleles associated with this genetic disease in “Allelic Variants”. Click on “Table View”. Do you find the variant **Cys2307Ser** our patient carries in the “Mutation” column of the Allelic Variants table? You can click on the hyperlink in the “ClinVar” column and see what is currently known about this particular sequence variant.

**Q5. Type your diagnosis for your patient based on his genetic testing result.**

Go back to the search results. Click on the OMIM record for your diagnosis (answer to Q5). This page shows the OMIM summary for this genetic condition. On the upper right corner, under the “External Links”, open Clinical Resources and click on “GeneReviews” This website summarizes practical information for a clinician to care for patients with this genetic condition. For example, “Management” under “Summary” provides recommendations for how these patients are treated.
