# Supplementary material for: A Case-Based Active Learning Session for Medical Genetics Resources
Source: MedEdPORTAL. 2021 Apr 1;17:11135. doi: 10.15766/mep_2374-8265.11135 (PMC8015619; doi:10.15766/mep_2374-8265.11135)
Supplement: Supplementary file 1 — Syllabus Introduction.docxStudent Preclass Hands-on Exercise.docxSession Timetable.docxDidactic In-class Discussion.docxStudents In-class Activity.docxFaculty Preclass Hands-on Exercise.docxFaculty Guide In-class Activity.docxPostsession Survey.docx [file mep_2374-8265.11135-s001.zip › C. Session Timetable.docx]

**Table. Detailed Timeline of Learning Activities for the Medical Genetics Resources Session**

| **Required activities** | | **Links & Resources** | **Time Required (Minutes)** | **Appendix** |
| --- | --- | --- | --- | --- |
| **Pre-Class Individual Activities** | | | | |
| On your own | Read session syllabus to learn about  •  Pre-class Assignment: OMIM Tutorial  •  Questions to ask for a patient with a potential genetic condition  •  Recommended medical genetics resources | “Evidence-based and Lifelong Learning in Human and Medical Genetics” [PDF posted to LMS] | 15 | A |
| On your own | Work through and submit answers to Pre-class Hands-on Exercise | “Pre-class Assignment - OMIM Tutorial for Single Gene Disorders” [PDF posted to LMS]  Submit answers using quiz function in LMS | 45 | B |
| **In-class Case-based Active Learning Session** | | | | |
|  | Students assemble in small groups and prepare to work:   - Students sign attendance sheet - Students locate their small groups - Students set up personal computers connected to Wi-Fi | Student sign-in sheet [print]  Small group assignment sheet [print + posted to LMS]  Note: The group assignment remains the same through the session |  |  |
| Didactic & large group discussion | - Pre-class activity debrief & discussion - Expanding on pre-class activity, live demonstration of external links in OMIM | Review answer submissions for Pre-class Hands-on Exercise    Demo based on “Didactic In-class Discussion” document | 5  10 | D |
| Small groups | Work through first set of case questions:   - Draw family pedigree - Find answers/discuss answers to questions 1-3 | In-class Case-based Group Learning Activity, Part 1 [Part 1 PDF posted on LMS at the start of the session. Do not release Part 2 PDF until later in the session.]    Refer to session syllabus: “Evidence-based and Lifelong Learning in Human and Medical Genetics” [PDF posted on LMS]    Paper and pencils for creating pedigrees | 25 | E  G  (facilitators refer to the facilitator’s guide) |
| Large group | Debrief: In-class Case-based Group Learning Activity, Part 1   - Q&A: Groups selected by lottery to share answers |  | 15 |  |
| Brief didactic | Presentation by genetic counsellor on the role of the genetic care and collaboration with physicians in management of patients with genetic conditions | If participation of a genetic counselor is not possible, general information about genetic counselors can be found at the National Society of Genetic Counselors website: https://www.nsgc.org/page/aboutgeneticcounselors | 10 | G  (Brief description is included in the faculty version Appendix G) |
| Small groups | Work through second set of case questions   - Review genetic test results - Find/discuss answers to questions 4-8 | In-class Case-based Group Learning Activity, Part 2  [PDF posted on LMS – do not release Part 2 content until this section begins] | 25 | E  G  (facilitators refer to the facilitator’s guide) |
| Large group | Debrief: In-Class Case-based Group Learning Activity, Part 2   - Q&A: Groups selected by lottery to share answers |  | 20 |  |

LMS: Learning Management System
