# Supplementary material for: A Case-Based Active Learning Session for Medical Genetics Resources
Source: MedEdPORTAL. 2021 Apr 1;17:11135. doi: 10.15766/mep_2374-8265.11135 (PMC8015619; doi:10.15766/mep_2374-8265.11135)
Supplement: Supplementary file 1 — Syllabus Introduction.docxStudent Preclass Hands-on Exercise.docxSession Timetable.docxDidactic In-class Discussion.docxStudents In-class Activity.docxFaculty Preclass Hands-on Exercise.docxFaculty Guide In-class Activity.docxPostsession Survey.docx [file mep_2374-8265.11135-s001.zip › D. Didactic In-Class Discussion.docx]

**Didactic In-class Discussion**

**Explore the OMIM page for Marfan syndrome and the content found under External Links**

1. Let’s consider the risk of this patient’s relatives being affected with this genetic disease. Look for the information for the mode of inheritance and the penetrance of this genetic disease. Assess the risks for 1^st^-degree and 2^nd^-degree relatives. (Hint: Refer to the Marfan syndrome OMIM Phenotype-Genotype Relationship table, the inheritance column. GeneReviews offers more comprehensive information, i.e. Genetic Counseling for the patient and his family)

**(Answer)**

The mode of inheritance for Marfan syndrome is autosomal dominant with variable expressivity. Marfan syndrome’s penetrance is thought to be high.

First-degree relatives of the patient have a 50% chance and second-degree relatives, a 25% chance, of being affected.

1. Are any clinical trials available for a patient with this genetic condition? Where can you find the information? (Hint: look under Clinical Resources under ‘External Links’)

**(Answer)**

Access the “ClinicalTrials.gov” website (under External Links) from OMIM Marfan syndrome page to find information about Marfan syndrome clinical trials.

1. Where can you find information for patient support groups? (Hint: helpful sites, GARD, MedlinePlus Genetics)

**(Answer)**

On the GARD Marfan syndrome webpage, click on “Organizations” hyperlink to obtain the list of patient support groups. On MedlinePlus Genetics Marfan syndrome webpage, patient resources are listed under Additional Information & Resources.
