# Supplementary material for: A Case-Based Active Learning Session for Medical Genetics Resources
Source: MedEdPORTAL. 2021 Apr 1;17:11135. doi: 10.15766/mep_2374-8265.11135 (PMC8015619; doi:10.15766/mep_2374-8265.11135)
Supplement: Supplementary file 1 — Syllabus Introduction.docxStudent Preclass Hands-on Exercise.docxSession Timetable.docxDidactic In-class Discussion.docxStudents In-class Activity.docxFaculty Preclass Hands-on Exercise.docxFaculty Guide In-class Activity.docxPostsession Survey.docx [file mep_2374-8265.11135-s001.zip › E. Students In-Class Activity.docx]

**In-class Case-based Group Learning Activity**

**Part 1**

A 27-year-old female (Ms. L) came to the clinic with a concern for her risk of developing cancer because of her family history. She was also born with a cleft lip and palate, which were considered to be non-syndromic at birth. Otherwise, her personal health history is unremarkable. Her family history is as follows:

Her father was diagnosed with terminal stomach cancer at 33-years-old and passed away 5 months later. Her paternal grandmother and paternal great-grandmother were diagnosed with breast cancer at 42-years-old and 50-years-old, respectively. They both passed away shortly after diagnosis. Her 55-year-old paternal aunt has never had cancer. She has a 25-year-old brother who is healthy. Her maternal side of the family history is unremarkable. Neither side of her family are of Ashkenazi Jewish descent.

She is concerned about the possible hereditary cancer risk in her family and requests appropriate referral to further examine this.

**Q1.** Draw a pedigree of Ms. L’s family. Based on her family history, are you concerned that the patient is at risk for a hereditary cancer syndrome?

**Q2.** Based on her personal and family histories, propose candidate diagnoses for Ms. L’s family. You can utilize the recommended medical genetics database websites in the syllabus (Appendix A), or other resources you find reliable (document the name and URLs for those websites and describe the reason why you have concluded they are reliable). As a group, discuss which online resources you have selected and the search terms you have used.

**Q3.** Ms. L is interested in pursuing genetic testing. Discuss as a group which genetic testing approach would be most indicated in this case (i.e. single gene testing, multi-gene panel, whole exome sequencing, whole genome sequencing)? Explore pros and cons of each approach. Do not worry about selecting/identifying a specific genetic test.

**Part 2**

Ms. L’s genetic test results came back with the following results:

| ***BRCA1/2 Analyses*** |
| --- |
| **RESULTS** |
| *CDH1*  **Pathogenic Mutation: c.480_486del CATCAGCInsAGAATA** |
| *SMARCA4* Variant, Unknown Significance: p.P159L |
| **SUMMARY** |
| **POSITIVE: Pathogenic Mutation Detected** |
| **INTERPRETATION** |
| This individual is heterozygous for the **c.480_486del CATCAGCInsAGAATA** pathogenic mutation in the *CDH1* gene.  This result is consistent with a diagnosis of hereditary diffuse gastric cancer (HDGC) syndrome.  **Risk estimate:** lifetime risks of 67-83% for diffuse gastric cancer and 39-52% for lobular breast cancer (females only).  The expression and severity of disease for this individual cannot be predicted.  Genetic testing for pathogenic mutations in family members can be helpful in identifying at-risk individuals.  Genetic counseling is a recommended option for all individuals undergoing genetic testing. |

Her attending physician is responsible for discussing how to manage her health based on the test results. This is a collaborative endeavor with the genetic counselor. Discuss the following as a group.

**Q4.** Explain why the identified variant was classified to be pathogenic.

**Q5.** As an individual with this pathogenic variant, what is her risk of developing cancer? How about her brother? How about her future children? Can we be sure that her cleft palate and cleft lip are truly non-syndromic? (OMIM and GeneReviews are useful resources)

**Q6.** Ms. L wants to know where she can find more information and support for her and her family. Find information for patient support groups for her and discuss what types of support they each offer.

**Q7.** Her brother is also interested in being tested for the *CDH1* variant. Currently, he is applying for jobs and concerned about discrimination based on his genetic information. (genetic discrimination - <https://medlineplus.gov/genetics/understanding/testing/discrimination/>) He wants to know what legal protections he can expect should he test positive for the pathogenic mutation. Provide the information and explain the types and limits of protections for him. (Useful resource - <http://ginahelp.org/>, <https://www.genome.gov/about-genomics/policy-issues/Genetic-Discrimination>, https://www.dol.gov/agencies/ebsa/laws-and-regulations/laws/gina)

**Q8.** Are there any prophylactic procedures available for Ms. L? Explain the benefits and risks of this management approach. (Useful resources: GeneReviews, UpToDate, MedGen)
