# Supplementary material for: A Case-Based Active Learning Session for Medical Genetics Resources
Source: MedEdPORTAL. 2021 Apr 1;17:11135. doi: 10.15766/mep_2374-8265.11135 (PMC8015619; doi:10.15766/mep_2374-8265.11135)
Supplement: Supplementary file 1 — Syllabus Introduction.docxStudent Preclass Hands-on Exercise.docxSession Timetable.docxDidactic In-class Discussion.docxStudents In-class Activity.docxFaculty Preclass Hands-on Exercise.docxFaculty Guide In-class Activity.docxPostsession Survey.docx [file mep_2374-8265.11135-s001.zip › G. Faculty Guide In-class Activity.docx]

**Faculty Guide to the In-class Case-based Group Learning Activity**

**Part 1**

A 27-year-old female (Ms. L) came to the clinic with a concern for her risk of developing cancer because of her family history. She was also born with a cleft lip and palate, which were considered to be non-syndromic at birth. Otherwise, her personal health history is unremarkable. Her family history is as follows:

Her father was diagnosed with terminal stomach cancer at 33-years-old and passed away 5 months later. Her paternal grandmother and paternal great-grandmother were diagnosed with breast cancer at 42-years-old and 50-years-old, respectively. They both passed away shortly after diagnosis. Her 55-year-old paternal aunt has never had cancer. She has a 25-year-old brother who is healthy. Her maternal side of the family history is unremarkable. Neither side of her family are of Ashkenazi Jewish descent.

She is concerned about the possible hereditary cancer risk in her family and requests appropriate referral to further examine this.

**Q1.** Draw a pedigree of Ms. L’s family. Based on her family history, are you concerned that the patient is at risk for a hereditary cancer syndrome?


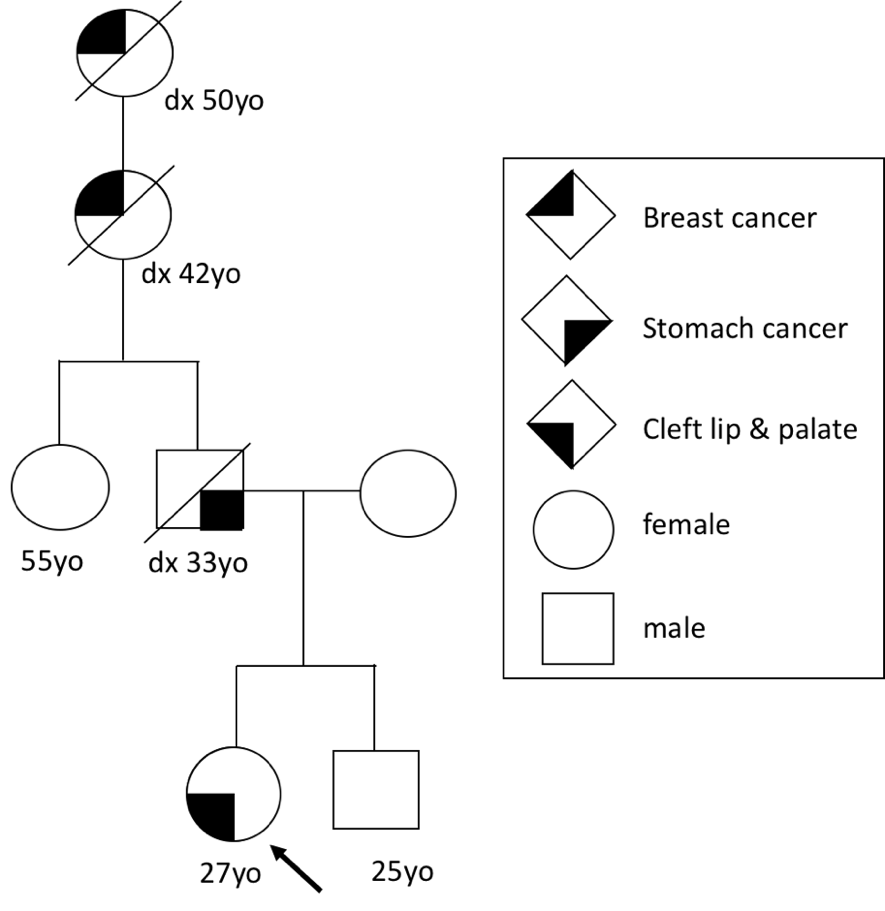


**(Pedigree)**

**(Answer)**

Yes. Her family history indicates that multiple individuals spanning 3 generations were diagnosed with cancer at 50 years old or younger. Therefore, the likelihood of a hereditary cancer syndrome affecting this family is very high. Even if you have not heard of the clustering of stomach cancer and breast cancer in a family affected by a cancer predisposition syndrome before, discovery of new genetic syndromes is ongoing and you should still consider referring to a genetic counselor for further evaluation.

**Q2.** Based on her personal and family histories, propose candidate diagnoses for Ms. L’s family. You can utilize the recommended medical genetics database websites in the syllabus (Appendix A), or other resources you find reliable (document the name and URLs for those websites and describe the reason why you have concluded they are reliable). As a group, discuss which online resources you have selected and the search terms you have used.

**For facilitators: Suggested search hints for students:**

- To encourage applying different combinations of search terms when using OMIM.
- To consider using a synonym for stomach cancer, i.e. gastric cancer.
- To make ‘breast cancer’ as an obligatory search term because of her family’s breast cancer history.
- To recommend using the “Clinical Synopsis” function to limit the results to phenotypic entries.
- To consider the possibility that Ms. L’s cleft palate and cleft lip might be part of the syndrome. New scientific discoveries may have been made since she was born.

**Examples of search term combinations students used at the session were:**

1. +’breast cancer’ ‘stomach cancer’ and **Clinical synopsis** at OMIM – Hereditary Breast and Ovarian Cancer syndromes (*BRCA2* and *BRCA1*) were the top 2 results, followed by Hereditary Diffuse Gastric Cancer (HDGC). (accessed on June 22, 2020)
2. +‘breast cancer’ ‘gastric cancer’, and **Clinical synopsis** at OMIM - Hereditary Diffuse Gastric Cancer (HDGC) was the first in the retrieved results, followed by Breast Cancer (multiple genes associated), *BRCA1* Hereditary Breast and Ovarian Cancer syndrome, and *BRCA2* Hereditary Breast and Ovarian Cancer syndrome. (accessed on June 22, 2020)
3. +’breast cancer’ +’stomach cancer’ and **Clinical synopsis** at OMIM – Hereditary Breast and Ovarian Cancer syndrome (*BRCA2* and *BRCA1*) were the top 2 results, followed by Hereditary Diffuse Gastric Cancer (HDGC). The number of the returned results are less than that obtained by the search terms used in (a). (accessed on June 22, 2020)
4. +’breast cancer’ +’gastric cancer’ and **Clinical synopsis** at OMIM – Hereditary Diffuse Gastric Cancer (HDGC) was listed first in the retrieved results, followed by Breast Cancer (multiple genes associated) and Lung Cancer (multiple genes associated). (accessed on June 22, 2020)
5. +’breast cancer’ ‘stomach cancer’ ‘cleft palate’ and **Clinical synopsis** at OMIM – Hereditary Diffuse Gastric Cancer (HDGC) was first in the retrieved results, followed by *BRCA2* Hereditary Breast and Ovarian Cancer syndrome and *BRCA1* Hereditary Breast and Ovarian Cancer syndrome. (accessed on June 22, 2020)
6. +’breast cancer’ +’gastric cancer’ +‘cleft palate’ at OMIM – Hereditary Diffuse Gastric Cancer (HDGC) was first in the retrieved results, followed by the Cadherin 1 gene (one of the HDGS associated genes) and the *FGFR2* gene. (accessed on June 22, 2020)

**Q3.** Ms. L is interested in pursuing genetic testing. Discuss as a group which genetic testing approach would be most indicated in this case (i.e. single gene testing, multi-gene panel, whole exome sequencing, whole genome sequencing)? Explore pros and cons of each approach. Do not worry about selecting/identifying a specific genetic test.

**(Answer)**

In this case, beginning with a breast cancer gene panel or multi-cancer gene panel is indicated since a similar multi-gene panel for stomach cancer is not currently available. Though the early age of onset and severity highly suggest a hereditary risk factor might be playing a role in her father’s stomach cancer, given the singular incidence in the family, we are still unsure about the risk it may pose for Ms. L at this time.

**Large Group Discussion**

- Question-Answer debrief for Part 1 Q1-3
- **Brief Didactic:** Genetic counselor informal presentation

The genetic counselor, who was one of the facilitators of the in-class session, provided a brief overview on the role of primary care/family medicine physicians and a genetic counselor, the process of referring the patient to a cancer genetic counselor, and the main components of a cancer genetic counseling session. If participation of a genetic counselor is not possible, general information about genetic counselors can be found at the National Society of Genetic Counselors website: <https://www.nsgc.org/page/aboutgeneticcounselors>

**After Brief Didactic, release Part 2 to students**

**Part 2**

Ms. L’s genetic test results came back with the following results:

| ***BRCA1/2 Analyses*** |
| --- |
| **RESULTS** |
| *CDH1*  **Pathogenic Mutation: c.480_486del CATCAGCInsAGAATA** |
| *SMARCA4* Variant, Unknown Significance: p.P159L |
| **SUMMARY** |
| **POSITIVE: Pathogenic Mutation Detected** |
| **INTERPRETATION** |
| This individual is heterozygous for the **c.480_486del CATCAGCInsAGAATA** pathogenic mutation in the *CDH1* gene.  This result is consistent with a diagnosis of hereditary diffuse gastric cancer (HDGC) syndrome.  **Risk estimate:** lifetime risks of 67-83% for diffuse gastric cancer and 39-52% for lobular breast cancer (females only).  The expression and severity of disease for this individual cannot be predicted.  Genetic testing for pathogenic mutations in family members can be helpful in identifying at-risk individuals.  Genetic counseling is a recommended option for all individuals undergoing genetic testing. |

Her attending physician is responsible for discussing how to manage her health based on the test results. This is a collaborative endeavor with the genetic counselor. Discuss the following as a group.

**Q4.** Explain why the identified variant was classified to be pathogenic.

**(Answer)**

This variant involves a 7 bp deletion and 6 bp insertion in the coding region of the *CDH1* gene at the nucleotide position 480-486 that leads to a frameshift at the 161^st^ amino acid of the e-cadherin protein. The full-length protein is 882 amino acids long and this frameshift mutation causes a premature truncation, therefore resulting in a nonfunctional protein.

**Q5.** As an individual with this pathogenic variant, what is her risk of developing cancer? How about her brother? How about her future children? Can we be sure that her cleft palate and cleft lip are truly non-syndromic? (OMIM and GeneReviews are useful resources)

**(Answer)**

Hereditary diffuse gastric cancer (HDGC) is an autosomal dominant cancer predisposition syndrome. *CDH1* is a tumor suppressor gene; those with a heterozygous pathogenic variant have a 70 to 80% lifetime risk of developing diffuse gastric cancer, attributed to loss of heterozygosity caused by inactivation of the remaining wild type CDH1 allele. In addition to gastric cancer, up to 60% of females develop lobular carcinoma of the breast. An association with colon and/or rectal cancer was also suggested in the past, but the risks are unclear and likely not increased based on information available at this time. (OMIM # 137215 HDGC; <https://www.cancer.net/cancer-types/hereditary-diffuse-gastric-cancer>, National Comprehensive Cancer Network Guidelines for Gastric Cancer, Ver 2.2020)

Since HDGC follows the autosomal dominant mode of inheritance, her brother has a 50% chance of also being positive for this pathogenic variant, and each of her future children have a 50% chance of inheriting this pathogenic variant from Ms. L.

*CDH1* variants were also recently discovered in association with cleft lip and palate formation, therefore suggesting the likelihood that Ms. L’s cleft palate and cleft lip may be syndromic in nature. Genetic underpinnings of human diseases are constantly updated, and as such, prior diagnoses should not be treated as immutable.

**Q6.** Ms. L wants to know where she can find more information and support for her and her family. Find information for patient support groups for her and discuss what types of support they each offer.

**(Answer)**

For example, search for “Hereditary Diffuse Gastric Cancer” at the GARD website. Under “Organizations”, you can find the list of patient family support groups. At the MedlinePlus Genetics website, patient support information can be found under the “Additional Information” tab.

In providing care for a patient with a genetic condition, collaborative management with genetic specialists (such as genetic counselors and medical geneticists) is critical. Maintaining an active partnership with them can help provide the best fitted care to your patient.

**Q7.** Her brother is also interested in being tested for the *CDH1* variant. Currently, he is applying for jobs and concerned about discrimination based on his genetic information. (genetic discrimination - <https://medlineplus.gov/genetics/understanding/testing/discrimination/> ) He wants to know what legal protections he can expect should he test positive for the pathogenic mutation. Provide the information and explain the types and limits of protections for him. (Useful resource - <http://ginahelp.org/>, <https://www.genome.gov/about-genomics/policy-issues/Genetic-Discrimination>, https://www.dol.gov/agencies/ebsa/laws-and-regulations/laws/gina)

**(Answer)**

Refer to the GINA (Genetic Information Nondiscrimination Act) websites. These websites provide information on federal level protection for individuals with genetic conditions.

The federal Genetic Information Nondiscrimination Act (GINA) protects individuals against life insurance and employment discrimination.

Current federal law prohibits the use of a genetic test result against an insured to deny or alter the health insurance premium (this does not apply to an individual with a manifested genetic disease condition). Additionally, it is illegal for employers to use a person's genetic information when making decisions about hiring, promotion, and several other terms of employment.

There are limitations of the protections provided by GINA.

Insurance Limitations:

- GINA does not protect against discrimination by life, disability, or long-term care insurance.
- GINA also does not prevent health insurers from determination of eligibility or premiums based on current symptoms/disease/diagnosis, even if the diagnosis was in part due to a genetic test.
- Current federal health insurance provisions do not apply to members of the U.S. military, nor individuals receiving medical service via the Veteran’s Administration, Indian Health Service, and Federal Employees Health Benefit Plans (although these plans have their own protections similar to that of GINA)

Employment:

- GINA does not prohibit discrimination by an employer that has fewer than 15 employees.
- GINA also does not apply to military and Federal employees. An executive order currently provides additional protection for federal employees, while the military has separate policies that may or may not provide sufficient genetic nondiscrimination protections.

Additional genetic information nondiscrimination protection varies widely by state. For example, California’s Genetic Information Nondiscrimination Act (CalGINA) extends protections to further prohibit genetic discrimination by housing, mortgage lending, emergency medical services, and other state-funded programs. Other states such as Mississippi and Pennsylvania, do not provide any additional nondiscrimination protection. Therefore, consultation with a local genetic counselor is imperative to ensure the patient’s awareness of potential consequences associated with genetic testing.

More details including limitations can be found at the following websites:

- Federal genetic discrimination protection: <https://www.genome.gov/about-genomics/policy-issues/Genetic-Discrimination>
- Statewide genetic discrimination protection: https://www.genome.gov/about-genomics/policy-issues/Genome-Statute-Legislation-Database

**Q8.** Are there any prophylactic procedures available for Ms. L? Explain the benefits and risks of this management approach. (Useful resources: GeneReviews, UpToDate, MedGen)

**(Answer)**

To address the risks associated with this cancer syndrome, you and the genetic counselor should discuss screening guidelines and preventive healthcare options for risk reducing with the patient, i.e., annual monitoring of stomach by endoscopy with specific biopsy criteria (Cambridge protocol), risk reducing total gastrectomy, annual mammograms and breast MRI, as well as risk reducing prophylactic mastectomy. This is a process of facilitating decision-making involving all parties including the patient.
