# Supplementary material for: A Case-Based Active Learning Session for Medical Genetics Resources
Source: MedEdPORTAL. 2021 Apr 1;17:11135. doi: 10.15766/mep_2374-8265.11135 (PMC8015619; doi:10.15766/mep_2374-8265.11135)
Supplement: Supplementary file 1 — Syllabus Introduction.docxStudent Preclass Hands-on Exercise.docxSession Timetable.docxDidactic In-class Discussion.docxStudents In-class Activity.docxFaculty Preclass Hands-on Exercise.docxFaculty Guide In-class Activity.docxPostsession Survey.docx [file mep_2374-8265.11135-s001.zip › H. Postsession Survey.docx]

Post-session Survey

Q1. Have you searched for information on genetic conditions before this session?

- Yes [continue to Q2]
- No [skip to Q3]

Q2. In the past, which of the resources listed below would you have used as your **first source** to gain information on genetic conditions? [select one]

- OMIM
- GeneReviews
- MedlinePlus Genetics
- UpToDate
- PubMed
- Wikipedia
- Google
- Other (please specify) ________________________________________________

Q3. After this session, which of the resources listed below will be your **first choice** to gain information on genetic conditions? [select one]

- OMIM
- GeneReviews
- MedlinePlus Genetics
- UpToDate
- PubMed
- Wikipedia
- Google
- Other (please specify) ________________________________________________

Q4. Respond to this statement:

After this session, I feel more confident in using medical genetics resources to investigate genetic conditions.

- strongly agree
- agree
- neutral
- disagree
- strongly disagree

Q5. Please provide a short narrative as to what you found helpful / useful as well as what you found not helpful / useful in this session. Also, let us know anything you thought about this session. [continue comments on back if necessary]

_________________________________________________________________________________________________

_________________________________________________________________________________________________

_________________________________________________________________________________________________

Thank you for completing this survey!
