# Supplementary material for: A Case-Based Active Learning Session for Medical Genetics Resources
Source: MedEdPORTAL. 2021 Apr 1;17:11135. doi: 10.15766/mep_2374-8265.11135 (PMC8015619; doi:10.15766/mep_2374-8265.11135)
Supplement: Supplementary file 1 — Syllabus Introduction.docxStudent Preclass Hands-on Exercise.docxSession Timetable.docxDidactic In-class Discussion.docxStudents In-class Activity.docxFaculty Preclass Hands-on Exercise.docxFaculty Guide In-class Activity.docxPostsession Survey.docx [file mep_2374-8265.11135-s001.zip › _Educational_Summary_Report_11135.pdf]

MedEdPORTAL® The AAMC Journal of Teaching and Learning Resources

Original Publication

Open Access

A Case-Based Active Learning Session for Medical Genetics Resources

Hana Anderson, PhD*, Amy C. Studer, RN, MSN, MSLIS, AHIP, Katharine N. Holm, PhD, Ayaka Suzuki, MS, CGC

*Corresponding author: hnanderson@ucdavis.edu

Abstract

Introduction: As the clinical applications of medical genetics and genomics continue to expand, nongenetics professionals increasingly find themselves in the position of managing patients with genetic conditions. To prepare medical students to handle this future practice demand, it is imperative that they obtain skills and confidence in utilizing credible medical genetics resources to care for patients with genetic conditions. To this end, we developed active learning materials to introduce first-year medical students to these resources. **Methods:** This approximately 2-hour session targeted first-year medical students (123 students) and combined flipped classroom and small-group collaborative case-based learning models. Students first completed a hands-on preclass exercise, which guided them in navigating the Online Mendelian Inheritance in Man website, and then attended an in-person small-group classroom activity, which provided the opportunity to apply information obtained from credible medical genetics resources to a patient case. At the conclusion of the classroom activity, students voluntarily completed an anonymous survey. **Results:** Results of student postsession surveys showed that, regardless of previous exposure to medical genetics resources, this session increased both confidence in skills and future intention to use medical genetics resources. **Discussion:** Since the majority of students were unfamiliar with using specialized medical genetics resources prior to this educational intervention, the session functioned as a practical introduction to these essential resources. We propose that equipping medical students with skills that support inquiry-oriented learning, particularly in the early stage of training, can cultivate the practice of lifelong learning in medical genetics.

Keywords
Case-Based Learning, Evidence-Based Medicine, Lifelong Learning, Medical Genetics, Basic Science, Flipped Classroom

Educational Objectives

By the end of this activity, learners will be able to:

1. Identify clinical questions relevant to a patient with a possible genetic condition, guided by an established framework.
2. Utilize credible, specialized medical genetics resources (e.g., Online Mendelian Inheritance in Man and GeneReviews) to acquire information related to a clinical genetics case.
3. Apply the information gathered using specialized medical genetics resources to answer various types of questions generated during a medical genetics case (e.g., differential diagnosis, genotype-phenotype relationships, genetic testing, patient management, patient advocacy and support, family counseling, and clinical trials).
4. Apply basic genetics concepts and tools to clinical genetics cases (e.g., phenotypic heterogeneity, allelic heterogeneity, locus heterogeneity, and family pedigree).
5. Describe the value of collaborations with genetic counselors in the care of patients and families with suspected genetic conditions.

Citation:
Anderson H, Studer AC, Holm KN, Suzuki A. A case-based active learning session for medical genetics resources. *MedEdPORTAL*. 2021;17:11135. https://doi.org/10.15766/mep_2374-8265.11135

Introduction

The application of genetics and genomic medicine to clinical practice has been steadily expanding, owing to rapid advances in the knowledge of the genetic basis for a broad range of human diseases and the increased use of genetic testing.[1] For example, the recent expansion in applications of genomic medicine in cancer diagnosis and treatment[2] and clinical utility of whole exome sequencing[3] epitomize the trajectory of genomic medicine in practice. However, the number of genetics professionals (e.g., medical geneticists, genetic counselors) is not keeping pace with the growing demand.[4,5] Increasingly, nongenetics professionals are placed in the position of providing

Copyright © 2021 Anderson et al. This is an open-access publication distributed under the terms of the Creative Commons Attribution license.

care for patients with genetic conditions, creating an urgent need for genetics curricula to adapt.[6-8] As the clinical importance of medical genetics continues to grow, there is a compelling need to educate today's health professions students with the essential knowledge and skills that will allow them to adapt to the fast-paced progress of genetics and genomic medicine.[9]

Considering that there are over 5,000 monogenic disorders[10] and the fields of genetics and genomic medicine are constantly evolving,[11] it is critical that clinicians be able to identify and effectively use credible medical genetics resources at the point-of-care. To this end, undergraduate medical genetics education should support the development of inquiry-oriented critical thinking skills and lifelong learning practice beyond the memorization of factual knowledge. Indeed, the Association of Professors of Human and Medical Genetics' Medical School Genetics Competencies address the importance of these skills as a component of practice-based learning and improvement. They propose that students should be able to: (1) use information technology to obtain reputable current information about genetics, and (2) demonstrate skills required to stay abreast of advances in genetics that relate to medical practice. To achieve these competencies, it is required that medical students become proficient in utilizing medical genetics resources, such as Online Mendelian Inheritance in Man (OMIM) and GeneReviews.[12]

At the University of California (UC) Davis School of Medicine, we observed that first-year medical students typically do not have prior exposure to these resources, thereby presenting a skills gap that needed to be addressed. To this end, we developed an active learning session to introduce first-year medical students to credible medical genetics resources. This learning session combined a preclass individual hands-on guided exercise and an in-class small-group collaborative learning activity. The preclass exercise was based on a hypothetical clinical vignette to guide students in learning basic navigation of the OMIM website. The in-class session was developed around a patient case, which allowed students to learn how to apply medical genetics resources in solving questions presented by real-life situations. Case-based learning has been proven to enhance students' learning by aiding them in linking theory to clinical practice.[13] The positive learning effects of using a case-based approach in preclinical curriculum to stimulate students' engagement has been clearly demonstrated.[14-16]

In medical education literature, a few learning modules combining online medical genetics resources and patient vignettes have been published.[17-19] Two studies from the Johns Hopkins School of Medicine described how incorporating asynchronous OMIM training modules into a preclerkship genetics course was regarded positively by students and was associated with increased confidence in clinical genetics skills.[17,20] In our session, we built on the basic formula used in these previous reports and significantly expanded the student learning experience as described above. Our work makes a unique contribution to the literature by offering preclerkship students highly structured learning materials for introducing medical genetics resources, thereby providing know-how of genetic investigation in a time-efficient manner. This module could serve as a foundation for further genetics skills training during clerkship, such as reported by Hoffman et al.[21] Either in conjunction with clerkship modules or as a standalone activity, the resource presented here will encourage students to use specialized medical genetics resources as a lifelong learning practice in clinical genetics.

Methods

Curricular Context

At UC Davis School of Medicine, preclinical courses are currently offered during the first 2 years, including foundational science courses covering basic science principles of human health (first year), followed by pathophysiology and organ-based courses (second year). Genetics instruction is part of the foundational sciences at UC Davis. Within our competency-based curriculum framework, the genetics course supports graduation milestones in the knowledge competency, such that students will be able to apply current fundamental genetics knowledge to problem solving, as well as identify credible medical genetics resources and critically appraise information in the practice of lifelong learning and evidence-based medicine (EBM).

We designed this resource for first-year medical students as the first genetics active learning session during the 2019-2020 academic year. To receive full credit for this learning session, students needed to complete the preclass hands-on exercise (half credit) and attend the in-class active learning session (half credit). Our goals were to provide students with the opportunity to become familiar with specialized medical genetics resources, so that they could utilize these resources to practice EBM. In lectures prior to this session, students learned basic genetics concepts, such as human genome structure, genetic diversity in human populations, cytogenetics, mode of inheritance, population genetics, molecular tools for genetic testing, and molecular bases of genetic diseases. Students also had an active learning session in their epidemiology course that introduced them to credible medical resources in the context of beginning EBM instruction.

Copyright © 2021 Anderson et al. This is an open-access publication distributed under the terms of the Creative Commons Attribution license.

Preclass Individual Activities

A week prior to the in-class session, we provided students with the session syllabus (Appendix A) and guided hands-on exercise assignment (Appendix B). The syllabus included a list of recommended medical genetics resources and a framework of clinical questions for patients with possible genetic conditions. The question framework was based on the list previously published by Diehl et al.[17] The goal of the preclass hands-on exercise was to familiarize students with specialized medical genetics resources, with an emphasis on OMIM. To this end, we constructed a clinical vignette of a hypothetical Marfan syndrome patient and five associated questions modeling after an OMIM tutorial exercise published by Diehl et al.[17] To support asynchronous learning, we supplemented the assignment with detailed instructions for the relevant search and analysis functions available on the OMIM website (Appendix B). Students completed the preclass exercise individually and submitted their answers to the genetics course website prior to the in-class session. The correct responses are included for facilitators in Appendix F.

In-Class Session

The in-class session was held in an active learning classroom with small-group tables, each equipped with a large monitor, but can also be convened in a lecture hall. We divided the class (123 students) into 18 groups of six to seven students, assigned in alphabetical order. The in-class session was 110 minutes long and the time allotted to each activity was summarized in Appendix C. When conducting this learning activity using small-group rooms, we recommend limiting the group size to a maximum of 10 students, if possible, to encourage student participation.

Supplemental didactic discussion: At the beginning of the in-class session, we spent approximately 10 minutes exploring the clinical resources external links accessible from the OMIM website (e.g., GeneReviews, ClinicalTrials, MedlinePlus Genetics).[22] The genetics course instructor led this free-form didactic portion using three questions provided in Appendix D.

Case-based group learning activity: The in-class session was based on the case of a patient diagnosed with hereditary diffuse gastric cancer caused by a germline pathogenic variant in the cadherin-1 gene (Appendix E). Students were encouraged to use the medical genetics resources listed in the syllabus (Appendix A) to answer eight case-related questions. The first three questions were made available at the beginning of the in-class session and students were given 25 minutes to draw a pedigree using the patient's family history, propose possible diagnoses based on the family history, and discuss the appropriate type of genetic testing for the patient. A question and answer period (15 minutes) immediately followed, during which we randomly selected a student group to present an answer. After the question and answer session, a genetic counselor (one of the facilitators) shared a 10-minute informal introduction (Appendix G) on the role of a genetic counselor and how physicians and genetic counselors collaborate to manage patients. The patient's genetic testing result and the remaining five questions were then made available to students on the course website. With this final set of questions, students interpreted the genetic testing result, and explored the implications of the diagnosis for the patient and her family as well as resources to support and answer patient's questions. Twenty-five minutes were allotted for working on these questions and 20 minutes for the question and answer session that followed.

Facilitation of the in-class session: Four facilitators were present and circulated the room including two preclinical basic science faculty (PhD with background in human genetics), one genetic counselor, and one medical librarian. Facilitators answered students' questions and provided pointers to keep students on track. The presence of a genetic counselor and a librarian allowed us to model interprofessional teamwork for students. In preparation for the session, facilitators reviewed the syllabus and faculty guides (Appendices A, F, and G), which were provided to them 1 week prior. We also had a 30-minute presession meeting to discuss any questions. The two question-and-answer sessions at the in-class session were led by the instructor of the genetics course, who was also one of the facilitators.

Surveys: We distributed anonymous survey sheets (Appendix H) to each table at the beginning of the in-class session and collected them after the session. We designed the survey questions to collect: (1) students' prior experience with medical genetics resources before the session and students' intended use of the resources after the session, (2) students' comfort levels in using newly introduced medical genetics resources at the conclusion of the session, and (3) narrative responses to the session as a whole.

Results

Preclass Hands-On Exercise

All 123 first-year students completed all five questions of the required preclass hands-on exercise however time logs for nine students were not fully recorded and, therefore, they were excluded from time calculation. The average time taken by students to finish the exercise was 32 ± 24 minutes ($n = 114$).

Copyright © 2021 Anderson et al. This is an open-access publication distributed under the terms of the Creative Commons Attribution license.

Students' answers indicated that they followed the instructions closely and navigated the OMIM site as intended. Five students gave answers other than Marfan syndrome; however, these alternatives were phenotypically similar connective tissue disorders such as Marfanoid-Progeroid-Lipodystrophy syndrome and Loeys-Dietz syndrome type 1.

Postsession Survey Results

The voluntary postsession survey response rate was 46% (57 of the 123 students). Of the respondents, 30 students indicated they had done online searches on genetic conditions before this session and 27 had not. Figure 1 summarizes the reported usage of online resources by students while searching for information about genetic conditions prior to and after this session. Among the students with prior search experience, the most frequently used online sites before the session were PubMed, UpToDate, and Google. Only a small fraction of these students indicated that they had used OMIM and/or GeneReviews. After the session, the vast majority of students, independent of the prior search experience, said their choice of online resource for searching genetic conditions would be OMIM.

To the Likert-scale (1 = *strongly disagree*, 5 = *strongly agree*) question, "After this session, I feel more confident in using medical genetics resources to investigate genetic conditions" most students with prior search experience (96%) responded *strongly agree* or *agree*, as did most students without prior search experience (89%; Figure 2). In narrative responses regarding the session, students commented positively on the: (1) effectiveness of the preclass hands-on exercise, (2) value of learning experience using a patient case, (3) usefulness of OMIM, and (4) value of interacting with a genetic counselor and a medical librarian as facilitators. The representative narrative excerpts were:

- "The preclass assignment was very helpful—I was not aware of lots of the more nuanced functions of OMIM like 'compare selected,' tables, etc."
- "The questions were realistic. I wish we could have more specific case-based work like this."
- "I loved the OMIM website—it's tough to navigate at first, but so cool when you get used to it— will definitely use in future. I have it bookmarked on my computer!"
- "I found it helpful to hear from the genetic specialist and search experts. It was nice having to navigate the website on our own. Interesting discussion questions; I liked thinking about all aspects of care, including useful legal and support info."

Critical comments suggested that the in-class session could be shortened and that the class cover more than one genetic condition; a representative excerpt was, "Timeframe was a little long. Could have been interesting to investigate more than one condition instead of one condition in depth."

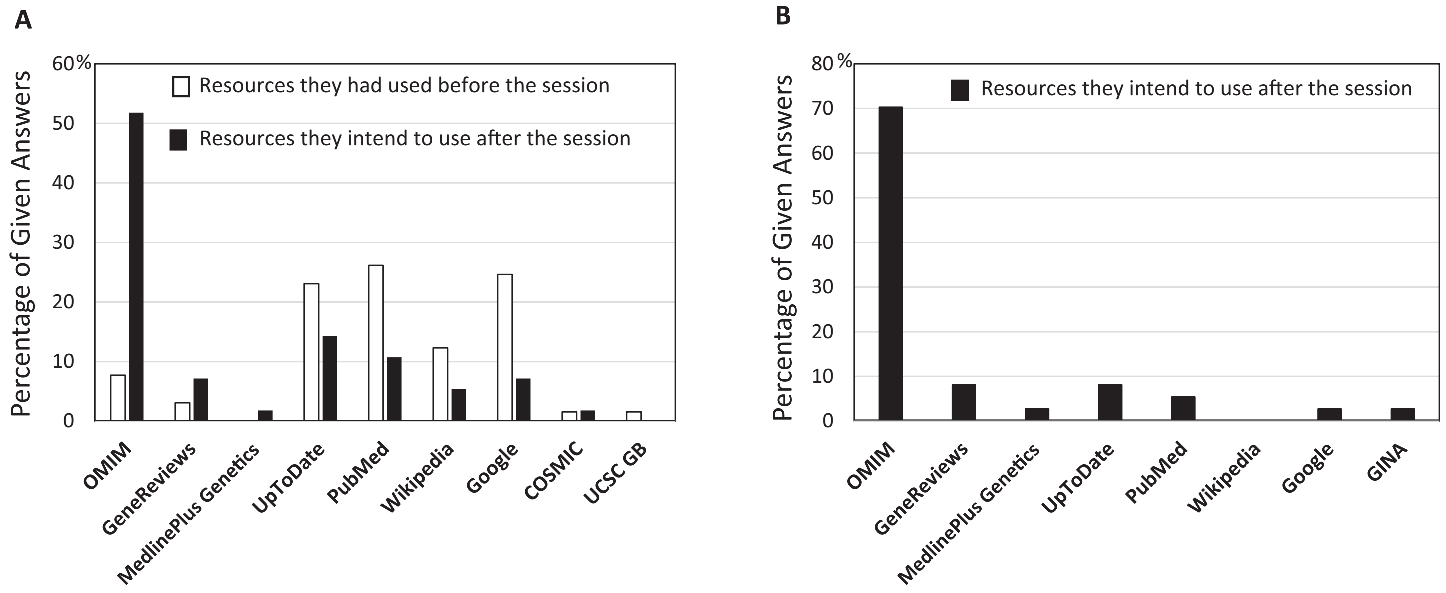

Figure 1. Pre- and postsession usage of online resources reported by 30 students with prior experience in online searching for genetic conditions (A) and 27 students without prior experience (B). Students could list more than one online resource.

Copyright © 2021 Anderson et al. This is an open-access publication distributed under the terms of the Creative Commons Attribution license.

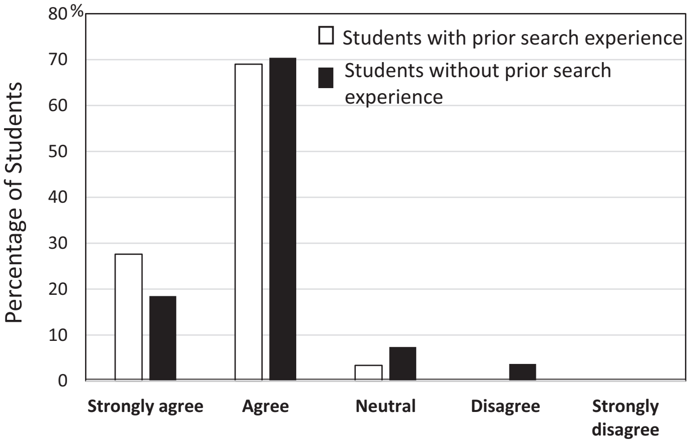

Figure 2. Postsession confidence levels in using medical genetics resources in the future. Twenty-nine students with prior search experience and 27 students without prior search experience responded to the question.

Discussion

We developed this session to provide practical knowledge and skills that enabled students to obtain reputable information on genetic conditions and apply it to clinical cases. We envisioned that the learning experience gained in the early stage of medical education will foster students' practice of EBM and lifelong learning in genetics and genomic medicine. The structure of our session was a combination of a flipped classroom and small-group collaborative case-based learning activity. At the end of the in-class session, we conducted a voluntary survey to assess perceived changes in attitudes, future intention to use medical genetic resources, and confidence levels in using these resources. Narrative survey responses helped to identify what worked and what can be improved. Having two opportunities to practice using the medical genetics resources—first as an individual assignment then as a group assignment—was received favorably by students. Specifically, they indicated that the preclass hands-on exercise oriented them in the basic functions of OMIM and the group activity allowed them to focus on obtaining germane information for solving the patient case. This was reflected by a narrative response such as, "I found utilizing these resources as a group was helpful, especially OMIM."

Our survey showed that about a half of the responding students had never searched for information on genetic conditions and the vast majority of them had never used OMIM before (Figure 1). Despite the fact that this session was their first encounter with OMIM, the students followed the hands-on exercise instructions closely and applied the learned skills successfully to the in-class patient case. In addition, regardless of prior search experience, a significant proportion of the students expressed their intention of using OMIM after the session (Figure 1) and increased confidence in medical genetics databases (Figure 2). The significant preference for OMIM demonstrated by students likely stemmed from the hands-on exercise which focused on the use of OMIM, even though we described the utility of other resources such as GeneReviews and ClinVar whose links are listed at the OMIM site as clinical resources. These results indicate that students become motivated to use specialized genetics resources when they acquired skills through active learning. We think adopting multiple instructional methods in the session also maximized learning by supporting various learning preferences of individual students.[23]

Lessons Learned and Implementation Challenges

We noticed that even though other useful resources such as GeneReviews were referred to in the students' materials (Appendices A, B, D, and E), they ranked low in the resource list of future intended use (Figure 1). This result revealed to us that tailoring exercise questions to different resources would be necessary to increase students' comfort level for using them. During the in-class session, progress varied among groups, leaving some fast-paced students unoccupied. This is a common challenge in dealing with multiple student groups and providing additional cases for extra credit could keep more advanced students engaged. Additional challenges with implementation may include recruiting a sufficient number of facilitators to support small-group learning.

Limitations

We have identified the following limitations in our analysis. First, results reported here were based on one class of students; therefore, conducting an analysis based on data from multiple classes of students would make our assessment more robust and reproducible. Second, observing the long-term effect of the session on students' behavior is desirable. Evaluating students' habits for medical genetics resources usage throughout the clinical curriculum would provide an appropriate outcome assessment. For example, at UC Davis, third-year students shadow genetic counselors counseling familial cancer cases during the OB/GYN clerkship. As Hoffman et al.[21] successfully delivered genetics cases during the third-year pediatrics clerkship, revisiting evidence-based genetics learning could provide a suitable opportunity to reevaluate and reinforce the use of medical genetics resources.

Future Directions

Currently, at UC Davis School of Medicine, we are redesigning our curriculum to be a patient- and learner-centered curriculum.

Copyright © 2021 Anderson et al. This is an open-access publication distributed under the terms of the Creative Commons Attribution license.

To enhance the clinical relevance of basic science education, it is important to teach students not simply factual knowledge, but also skills in identifying and validating current information so they can practice evidence-based patient care. To this end, we will continue to provide opportunities for student skill development in the effective use of medical genetics resources in the medical school curriculum. The resource presented here functions as an introductory module to essential skills for lifelong learning in managing patients with genetic conditions.

Appendices

A. Syllabus Introduction.docx

B. Student Preclass Hands-on Exercise.docx

C. Session Timetable.docx

D. Didactic In-class Discussion.docx

E. Students In-class Activity.docx

F. Faculty Preclass Hands-on Exercise.docx

G. Faculty Guide In-class Activity.docx

H. Postsession Survey.docx

All appendices are peer reviewed as integral parts of the Original Publication.

Hana Anderson, PhD: Associate Professor, Department of Internal Medicine and Department of Cell Biology and Human Anatomy, University of California Davis School of Medicine; ORCID: https://orcid.org/0000-0001-9159-1472

Amy C. Studer, RN, MSN, MSLIS, AHIP: Health Science Librarian, Blaisdell Medical Library, University of California Davis School of Medicine; ORCID: https://orcid.org/0000-0002-8852-6001

Katharine N. Holm, PhD: Research Associate, Department of Biochemistry and Molecular Medicine, University of California Davis

Ayaka Suzuki, MS, CGC: Licensed and Certified Genetics Counselor, Division of Genomic Medicine, Department of Pediatrics, University of California Davis

Disclosures
None to report.

Funding/Support
None to report.

Ethical Approval
Reported as not applicable.

References

1. Phillips KA, Deverka PA, Hooker GW, Douglas MP. Genetic test availability and spending: where are we now? Where are we going? *Health Aff (Millwood)*. 2018;37(5):710-716. https://doi.org/10.1377/hlthaff.2017.1427
2. Berger MF, Mardis ER. The emerging relevance of genomics in cancer medicine. *Nat Rev Clin Oncol*. 2018;15(6):353-365. https://doi.org/10.1038/s41571-018-0002-6
3. Farwell KD, Shahmirzadi L, El-Khechen D, et al. Enhanced utility of family-centered diagnostic exome sequencing with inheritance model-based analysis: results from 500 unselected families with undiagnosed genetic conditions. *Genet Med*. 2015;17(7):578-586. https://doi.org/10.1038/gim.2014.154
4. Cichon M, Feldman GL. Opportunities to improve recruitment into medical genetics residency programs: survey results of program directors and medical genetics residents. *Genet Med*. 2014;16(5):413-418. https://doi.org/10.1038/gim.2013.161
5. Stoll K, Kubendran S, Cohen SA. The past, present, and future of service delivery in genetic counseling: keeping up in the era of precision medicine. *Am J Med Genet C Semin Med Genet*. 2018;178(1):24-37. https://doi.org/10.1002/ajmg.c.31602
6. Burke S, Stone A, Bedward J, Thomas H, Farndon P. A “neglected part of the curriculum” or “of limited use”? Views on genetics training by nongenetics medical trainees and implications for delivery. *Genet Med*. 2006;8(2):109-115. https://doi.org/10.1097/01.gim.0000200159.19920.b5
7. Haspel RL, Saffitz JE. Genomic oncology education: an urgent need, a new approach. *Cancer J.* 2014;20(1):91-95. https://doi.org/10.1097/PPO.0000000000000015
8. Campion M, Goldgar C, Hopkin RJ, Prows CA, Dasgupta S. Genomic education for the next generation of health care providers. *Genet Med*. 2019;21(11):2422-2430. https://doi.org/10.1038/s41436-019-0548-4
9. Talwar D, Chen WJ, Yeh YL, Foster M, Al-Shagrawi S, Chen LS. Characteristics and evaluation outcomes of genomics curricula for health professional students: a systematic literature review. *Genet Med*. 2019;21(8):1675-1682. https://doi.org/10.1038/s41436-018-0386-9
10. Prakash V, Moore M, Yáñez-Muñoz RJ. Current progress in therapeutic gene editing for monogenic diseases. *Mol Ther*. 2016;24(3):465-474. https://doi.org/10.1038/mt.2016.5
11. Green ED, Gunter C, Biesecker LG, et al. Strategic vision for improving human health at the forefront of genomics. *Nature*. 2020;586(7831):683-692. https://doi.org/10.1038/s41586-020-2817-4
12. Hyland KM, Dasgupta S, Garber K, et al. *Medical School Core Curriculum in Genetics 2013.* Association of Professors of Human and Medical Genetics; 2013. Accessed December 11, 2014. http://media.wix.com/ugd/3a7b87_7064376a9eb346cfa1b85bc2f137c48f.pdf

Copyright © 2021 Anderson et al. This is an open-access publication distributed under the terms of the Creative Commons Attribution license.

13. Thistlethwaite JE, Davies D, Ekeocha S, et al. The effectiveness of case-based learning in health professional education. A BEME systematic review: BEME guide no. 23. *Med Teach*. 2012;34(6): e421-e444. https://doi.org/10.3109/0142159X.2012.680939

14. Bean LJ, Fridovich-Keil J, Hegde M, Rudd MK, Garber KB. The virtual diagnostic laboratory: a new way of teaching undergraduate medical students about genetic testing. *Genet Med*. 2011;13(11):973-977. https://doi.org/10.1097/GIM.0b013e318225ac13

15. Dickinson BL, Lackey W, Sheakley M, Miller L, Jevert S, Shattuck B. Involving a real patient in the design and implementation of case-based learning to engage learners. *Adv Physiol Educ*. 2018;42(1):118-122. https://doi.org/10.1152/advan.00174.2017

16. Anderson H, Sweeney C, Perry R, Vaquero J, Ison H. Patient-centered team-based learning in preclinical curriculum supporting the application of knowledge to real-world clinical experience. *Med Sci Educ*. 2020;30:65-68. https://doi.org/10.1007/s40670-019-00872-5

17. Diehl AC, Reader L, Hamosh A, Bodurtha JN. Horizontal integration of OMIM across the medical school preclinical curriculum for early reinforcement of clinical genetics principles. *Genet Med*. 2015;17(2):158-163. https://doi.org/10.1038/gim.2014.84

18. Magee J, Gordon JI, Whelan A. Bringing the human genome and the revolution in bioinformatics to the medical school classroom: a case report from Washington University School of Medicine. *Acad Med*. 2001;76(8):852-855. https://doi.org/10.1097/00001888-200108000-00022

19. Waggoner DJ, Martin CL. Integration of internet-based genetic databases into the medical school preclinical and clinical curriculum. *Genet Med*. 2006;8(6):379-382. https://doi.org/10.1097/01.gim.0000223543.63104.5a

20. Lee-Barber J, Kulo V, Lehmann H, Hamosh A, Bodurtha J. Bioinformatics for medical students: a 5-year experience using OMIM in medical student education. *Genet Med*. 2019;21(2): 493-497. https://doi.org/10.1038/s41436-018-0076-7

21. Hoffman JD, Thompson R, Swenson KB, Dasgupta S. Complexities of clinical genetics consultation: an interprofessional clinical skills workshop. *MedEdPORTAL*. 2020;16:10869. https://doi.org/10.15766/mep_2374-8265.10869

22. Online Mendelian Inheritance in Man. Updated February 10, 2021. Accessed February 11, 2021. https://omim.org

23. Knowles MS. *The Modern Practice of Adult Education: From Pedagogy to Andragogy*. Association Press; 1980.

Received: August 14, 2020
Accepted: February 1, 2021
Published: April 1, 2021

Copyright © 2021 Anderson et al. This is an open-access publication distributed under the terms of the Creative Commons Attribution license.
